# The Influence of *Moringa Oleifera* Leaf Powder on Body Weight MYF5 and IGF‐1gene Expression in Japanese Quails

**DOI:** 10.1002/vms3.70400

**Published:** 2025-05-13

**Authors:** Kadriye Kurşun

**Affiliations:** ^1^ Faculty of Agriculture Department of Animal Science Çukurova University Adana Türkiye

**Keywords:** growth performance, IGF‐1 gene, Japanese quail, *Moringa oleifera*, MYF5 gene, sustainable poultry nutrition

## Abstract

This study investigates the effects of *Moringa oleifera* leaf powder (MOLP) supplementation on growth performance and the expression of MYF5 and IGF‐1 genes in Japanese quails. A total of 116 quails were assigned to four dietary treatments: Control, Moringa‐1 (2% MOLP), Moringa‐2 (4% MOLP), and Moringa‐3 (6% MOLP). Body weights were recorded weekly, and gene expression levels were analysed using quantitative real‐time PCR. The results showed significant improvements in body weight and feed efficiency in the Moringa‐2 and Moringa‐3 groups compared to the Control group (*p* < 0.05). Additionally, the expression levels of MYF5 and IGF‐1 increased dose‐dependent, with the highest expression observed in the Moringa‐3 group. These findings demonstrate the potential of MOL as a natural feed additive to enhance growth performance and modulate molecular pathways related to muscle development in poultry. This study provides valuable insights for sustainable poultry production and highlights the need for further research on optimal inclusion levels and the long‐term effects of MOL supplementation.

## Graphical abstract

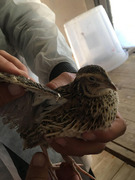

## 1 Introduction

The production of poultry eggs and meat has gained substantial interest due to their nutritional benefits, price, and the lack of any religious or traditional condemnation (Kursun et al., 2024a; Kurşun et al., 2024b; Baylan et al., 2024). Moreover, the recent rise in the global economic crisis, especially in developing and underdeveloped countries, has increased the global demand for chicken eggs (Abdallah et al. 2022; Baylan et al. 2024) and meat as the main source of animal protein. Additionally, the demand for poultry products, especially eggs, has increased tremendously due to increasing levels of income and standard of living (Pomaah et al. 2023). This has led to an increased interest in the production of other alternative poultry species such as quails for both eggs and meat (Ozkan et al. 2024). Japanese quails (*Coturnix japonica*) are widely recognised as a valuable model organism in poultry research due to their rapid growth, high reproductive efficiency, and adaptability to diverse environmental conditions (Huss et al. 2008; Ainsworth et al. 2010; Baer et al. 2015; Lukanov and Pavlova 2020). Their smaller size and shorter lifecycles make them suitable for experimental studies, enabling researchers to investigate the genetic, nutritional and environmental factors influencing their growth and development. In addition to their research significance, Japanese quails are economically important as a source of high‐quality meat and eggs, further highlighting the need to optimise their production performance (Carvalho et al. 2020; Zhu et al. 2021; Batool et al. 2023).

The growth performance of farm animals is a combined effect of the interaction of their genetic make‐up and their environment. Indicating the type of feed or composition of feed provided to the animal could influence the pathways and mechanisms involved in the activation of body growth and development. In addition, Musselman (2020) also reported that the genetic code or genotype of an animal is linked to different traits, and the interaction between the genotype and the environment can make a difference in performance. For instance, in Japanese quails, genetic pathways regulating muscle development play a pivotal role. Two critical genes, MYF5 and Insulin‐like Growth Factor‐1 (IGF‐1), have been identified as major regulators of muscle differentiation and growth, making them prime targets for studies aiming to enhance poultry performance. The MYF5 gene, a member of the myogenic regulatory factor (MRF) family, is essential in initiating myogenesis during embryonic development. As the first gene activated in the MRF family, which also includes MyoD, myogenin, and MRF4, MYF5 is crucial for determining the fate of myoblasts and promoting skeletal muscle fibre formation. Specific muscle‐related transcription factors, including MRFs (MYF5, MYOD, myogenin, and MRF4), PAX7, and PAX3, control myogenesis, and these factors act as terminal influencers of signalling procedures and contribute to the proper development of each stage‐specific transcript (Mohammadabadi et al. 2021). Additionally, PAX7 keeps satellite cells quiescent and, along with the expression of MYF5, plays a critical role in the development of activated myoblasts (Knight and Kothary 2011; Ridgeway and Skerjanc 2001). It was reported that MYOD and MYF5 are essential for the formation of muscle cell types, whereas myogenin and MRF4 are needed to stimulate differentiation and muscle fibre construction (Rehfeldt et al. 2011). Moreover, MYF5, MYOD and MRF4 are typically responsible for activating quiescent muscle stem cells and stimulating the genes required for muscle stem cell proliferation (Meadows et al. 2008).

The activity of MYF5 can be influenced by external factors such as diet and environmental conditions, suggesting its potential as a genetic marker for growth optimisation in poultry production (Fadhil and Zülkadir 2021; Bayraktar 2022; Bayraktar and Sezer 2022). IGF‐1 is traditionally recognised as a mediator of growth hormone activity; however, its regulation is multifactorial. In addition to being stimulated by growth hormone, IGF‐1 expression is also modulated by nutritional status, cytokine signalling, and local autocrine/paracrine mechanisms (Laron 2001; Bailes and Soloviev 2021). These additional regulatory pathways play crucial roles in controlling cellular proliferation, differentiation, and muscle hypertrophy, thereby contributing to overall growth and metabolic efficiency (Huat et al. 2014; Hawkes and Grimberg 2015; Yoshida and Delafontaine 2020; Youssef et al. 2024). Unlike MYF5, which is predominantly expressed in skeletal muscle, IGF‐1 is expressed in various tissues, including the liver, brain, and heart, making it a versatile candidate for studying dietary and hormonal influences on growth. Together, MYF5 and IGF‐1 represent key genetic pathways that can be modulated to enhance muscle development and production efficiency in poultry (Shit et al. 2014; Shafik et al. 2022; Ahmed et al. 2025).

One promising approach to optimising these genetic pathways is the use of natural feed supplements such as *Moringa oleifera* (MO), a plant known for its exceptional nutritional and bioactive properties (Mahfuz and Piao 2019; Mahmoud et al. 2019; Zhang et al. 2020). Often referred to as the ‘miracle tree’, *Moringa oleifera* is native to South Asia and parts of Africa and has long been used in human nutrition and traditional medicine (Mahfuz and Piao 2019; Shen et al. 2022). In recent years, its potential as a natural feed additive for livestock and poultry has garnered significant attention. *Moringa oleifera* leaves are particularly rich in essential nutrients, including proteins, vitamins and minerals, making them an ideal supplement for improving poultry diets. With a crude protein content of 17–22% (dry matter basis) and a rich amino acid profile including methionine, lysine and tryptophan, *Moringa oleifera* leaves provide a highly nutritious and balanced dietary supplement (Mahmud et al. 2016; Nwogor and Ifeyinwa 2017; Sati et al. 2021; Mulaudzi et al. 2022). In addition to its nutritional value, *Moringa oleifera* contains bioactive compounds such as flavonoids, phenolic acids and saponins, which exhibit antioxidant, anti‐inflammatory, and antimicrobial properties (Garcia et al. 2021; Shen et al. 2021; Imtiaz et al. 2023). These compounds help mitigate oxidative stress, which impairs muscle growth and overall health in poultry. By scavenging reactive oxygen species (ROS), the antioxidants in *Moringa oleifera* protect muscle cells from damage, promoting better growth and differentiation. Furthermore, its anti‐inflammatory properties may enhance immune function, improving health and productivity (Briones et al. 2015; El‐Kashef et al. 2017; Ahmed and El‐Rayes 2019; Atuahene et al. 2020). Despite its numerous benefits, *Moringa oleifera* also contains certain anti‐nutritional factors (Boğa et al. 2024), such as tannins, phytates and oxalates, which can interfere with nutrient absorption and metabolism, resulting in negative effects on growth performance and overall health of animals (Boga et al. 2024; Woyengo et al. 2017). For instance, it was reported that antinutritional factors such as phytic acids in their natural state form bonds with amino acids in protein bodies (Joyce et al. 2005; Lin et al. 2005; Ockenden et al. 2004), which is capable of minimising the availability of these nutrients for digestion and absorption (Lenis and Jongbloed 1999).

However, these components can be neutralised through processing methods such as drying, soaking, or fermentation, ensuring the plant's nutritional and bioactive potential is maximised. Understanding the dose‐dependent effects of *Moringa oleifera* is crucial, as excessive inclusion levels may negatively impact feed intake and growth performance due to these anti‐nutritional factors (Atuahene et al. 2019; El‐Son et al. 2022; Jalil and Yurtseven 2023). The potential of *Moringa oleifera* to influence gene expression, particularly in growth‐related genes such as MYF5 and IGF‐1, represents an exciting area of research. Although previous studies have shown that *Moringa oleifera* improves growth performance, immune function, and feed conversion efficiency in poultry, the molecular mechanisms underlying these effects remain poorly understood. This knowledge gap is particularly evident in dose‐dependent effects, where varying levels of *Moringa oleifera* supplementation may differentially affect gene expression in muscle development. Addressing these gaps is critical for developing targeted nutritional strategies that optimise poultry growth and production. Japanese quails provide an ideal model for investigating the effects of *Moringa oleifera* on gene expression due to their rapid growth rates, ease of management, and physiological similarities to other poultry species.

By studying the impact of *Moringa oleifera* supplementation on MYF5 and IGF‐1 expression in quails, researchers can gain valuable insights into the genetic and nutritional factors driving muscle development. These findings can then be translated into practical recommendations for improving growth performance in quails and other poultry species. Research has focused more on the influence of *Moringa oleifera* on live weight and feed intake with inadequate data on how this plant material interacts with genes involved in improving body growth in Japanese quails.

This study therefore aims to evaluate the dose‐dependent effects of *Moringa oleifera* supplementation on the mRNA expression of MYF5 and IGF‐1 in Japanese quails. By addressing the research gaps surrounding the interactions between *Moringa oleifera* and these key genes, the study seeks to provide a comprehensive understanding of how this natural feed additive influences muscle development and growth.

## 2 Materials and Methods

### 2.1 Ethics Statement

This study was conducted under the guidelines for animal experiments of the Ministry of Food, Agriculture and Livestock, Türkiye. Approval was granted by the local ethics committee of Cukurova University **(Approval number: No 1/31.01.2025)**.

### 2.2 Experimental Birds

This study was conducted at Çukurova University, Faculty of Agriculture, Research and Application Farm, Poultry Unit. The animal material consisted of 116 mixed‐sex day‐old Japanese quail chicks randomly assigned to four treatment groups: Control, Moringa‐1 (2% *Moringa oleifera* leaf powder (MOLP)), Moringa‐2 (4% MOLP), and Moringa‐3 (6% MOLP). Each treatment group consisted of 29 birds. Chicks were individually tagged on day one, and their live weights were recorded weekly throughout the 5‐week trial period. Weighing was performed individually using a precision scale with an accuracy of 0.1 g.

### 2.3 Feed Material

During the 5‐week growth period, the control group was fed a chick starter diet containing 24% crude protein and 3000 kcal/kg ME (0–2 weeks), followed by a broiler finisher diet containing 22% crude protein and 3000 kcal/kg ME (3–5 weeks). In the treatment groups, MOLP was added to the conventional diet at different inclusion levels: 2% in Moringa‐1, 4% in Moringa‐2, and 6% in Moringa‐3. Feed and water were provided ad libitum throughout the trial. The physical and chemical composition of the MOLP used in the study is presented in Table 1.

**TABLE 1:** Physical and chemical composition of MOLP used in this study.

Property	Value	Unit
Moisture	7.88	%
Protein	22.78	g/100g
Carbohydrates	31.16	g/100g
Glucose	3.196	g/100g
Fructose	3.841	g/100g
Sucrose	0.000	g/100g
Fibre	10.02	g/100g
Total Antioxidant Capacity	478.43	mM Trolox/g
Total Phenolic Content	10.39	mg/g
Organic Acids		
Ascorbic acid	127.02	mg/100g
Citric acid	3.25	mg/100g
Malic acid	3.50	mg/100g
Oxalic acid	5.61	mg/100g
Tartaric acid	4.70	mg/100g
Colour Values		
L*	56.55	—
a*	−4.17	—
b*	25.82	—
Chroma (C*)	26.15	—
Hue	99.18	—
Minerals		
Ca	1.9000	g/100g
K	1.3900	g/100g
Mg	0.7697	g/100g
S	0.4607	g/100g
P	0.2388	g/100g
Si	0.2029	g/100g
Cl	0.1438	g/100g
Al	62.3300	mg/100g
Fe	47.2400	mg/100g
Mn	6.3560	mg/100g
Cr	5.7550	mg/100g
Ti	3.2990	mg/100g
Ni	1.9760	mg/100g
Zn	1.8350	mg/100g

### 2.4 Experimental Units

The quails were reared in a cage system from one day old until slaughter. During the first two weeks, chicks were kept in brooder cages, and from the second week onwards, they were transferred to grower cages. The grower cages measured 28 cm × 92 cm × 44 cm (height × length × width). Each experimental unit was equipped with four nipple drinkers and feeders extending along the length of the cage. A 24‐h light regimen combining natural and artificial lighting was applied throughout the study. Ventilation in the poultry house was maintained using a curtain system and a fan located at the end of the building.

### 2.5 Molecular Analysis

#### 2.5.1 Sample Collection and RNA Isolation

Breast muscle samples were collected from each quail at the end of the 5‐week feeding trial. Total RNA was isolated from the samples using the agMAX mirVana Total RNA Isolation Kit (Thermo Fisher Scientific, USA), following the manufacturer's protocol. The purity and concentration of RNA were determined using a spectrophotometer.

#### 2.5.2 cDNA Synthesis

According to the manufacturer's instructions, complementary DNA (cDNA) was synthesised from 4 µL of total RNA using the RevertAid First Strand cDNA Synthesis Kit (Thermo Fisher Scientific, USA). The reaction mixture included 4 µL of 5× reaction buffer, 1 µL of reverse transcriptase, and 11 µL of RNase /DNase‐free water, resulting in a final reaction volume of 20 µL. The thermal cycler programme for reverse transcription was set to 25°C for 10 min, 42°C for 15 min, and a final hold at 4°C. The synthesised cDNA's quality was verified by amplifying the housekeeping gene β‐actin, and cDNA samples were stored at −20°C until further analysis.

#### 2.5.3 Quantitative Real‐Time Polymerase Chain Reaction (qRT‐PCR)

Quantitative real‐time PCR (qRT‐PCR) was performed to quantify the expression levels of MYF5 and IGF‐1 genes using SYBR Green chemistry (Thermo Fisher Scientific, USA). The MYF5 gene had a product size of 170 bp, amplified using the forward primer ATGGAGGTGATGGACAGC and reverse primer ATGTGCTCCTCTTCCTCA. The IGF‐1 gene had a product size of 154 bp, amplified using the forward primer CTGTTTCCTGTCTACAGT and reverse primer GATGTGAGATGTTGAGAG. The housekeeping gene β‐actin, with a product size of 167 bp, was used as the normalisation control and amplified using the forward primer TGACCGCGGTACAAACACAG and reverse primer CATACCAACCATCACACCCTGA (Vitorino Carvalho et al. 2019). These primers and conditions ensured precise and reliable amplification for gene expression analysis. PCR reactions were conducted in a final volume of 20 µL, containing 10 µL of SYBR Green Master Mix, 1 µL of forward primer, 1 µL of reverse primer, 2 µL of cDNA and 6 µL of RNase‐free water. Amplification was performed in a qPCR thermal cycler under the following conditions: initial denaturation at 95°C for 5 min, followed by 35 cycles of denaturation at 95°C for 40 s, annealing at 60°C for 45 s, and extension at 72°C for 45 s. A final extension was performed at 72°C for 10 min. The housekeeping gene β‐actin was used as the normalisation control. Relative mRNA expression levels were calculated using the 2⁻ΔΔCt method and reported as fold changes.

### 2.6 Statistical Analysis

All experimental data were analysed using one‐way analysis of variance (ANOVA) with the Statistical Analysis System (Minitab 18). The normality of the data was assessed using the Shapiro–Wilk test prior to analysis. Results are expressed as mean ± standard error of the mean (SEM), and significance was determined at p < 0.05. Post hoc comparisons among treatment groups were performed using Duncan's multiple‐range test. The qRT‐PCR data were analysed using GraphPad Prism software version 6 (GraphPad Software, La Jolla, CA, USA).

## 3 Results

### 3.1 Growth Performance

The effects of MOLP supplementation on the growth performance of Japanese quails are summarised in Table 2. Body weight showed no significant differences among treatment groups at day 1 (*p* > 0.05) and week 1 (*p* > 0.05). However, starting from week 2, significant differences were observed (*p* < 0.05), with higher weights recorded in the Moringa‐2 and Moringa‐3 groups. At week 5, quails in the Moringa‐2 and Moringa‐3 groups exhibited significantly higher body weights (222.28 ± 4.75 g and 222.25 ± 6.49 g, respectively) than the Control and Moringa‐1 groups (Table 2).

**TABLE 2:** Effect of the MOLP supplementation on the growth performance.

Trait	Weeks	Dietary treatments				
		Control	Moringa‐1 (%2 MOL)	Moringa‐2 (%4 MOL)	Moringa‐3 (%6 MOL)	*p*‐value
Body weight	1 day	9.20 ± 0.15	8.83 ± 0.14	8.96 ± 0.15	9.21± 0.14	*p* > 0.05
	1 week	24.88 ± 0.86	25.44 ± 0.78	25.62 ± 0.99	24.03 ± 1.11	*p* > 0.05
	2 week	62.87 ± 1.70	63.73 ± 1.71	65.79 ±1.41	66.50 ± 1.90	*p* < 0.05
	3 week	120.55 ± 2.97	114.99 ± 3.33	120.61 ± 2.61	121.88 ± 3.26	*p* < 0.05
	4 week	171.20 ± 4.78	163.82 ± 6.22	174.75 ± 4.52	173.20 ± 4.42	*p* < 0.05
	5 week	223.36 ± 6.18	207.29 ± 6.84	222.28 ± 4.75	222.25 ± 6.49	*p* < 0.05

### 3.2 Expression Profiles of MYF5 and IGF‐1

**FIGURE 1:**
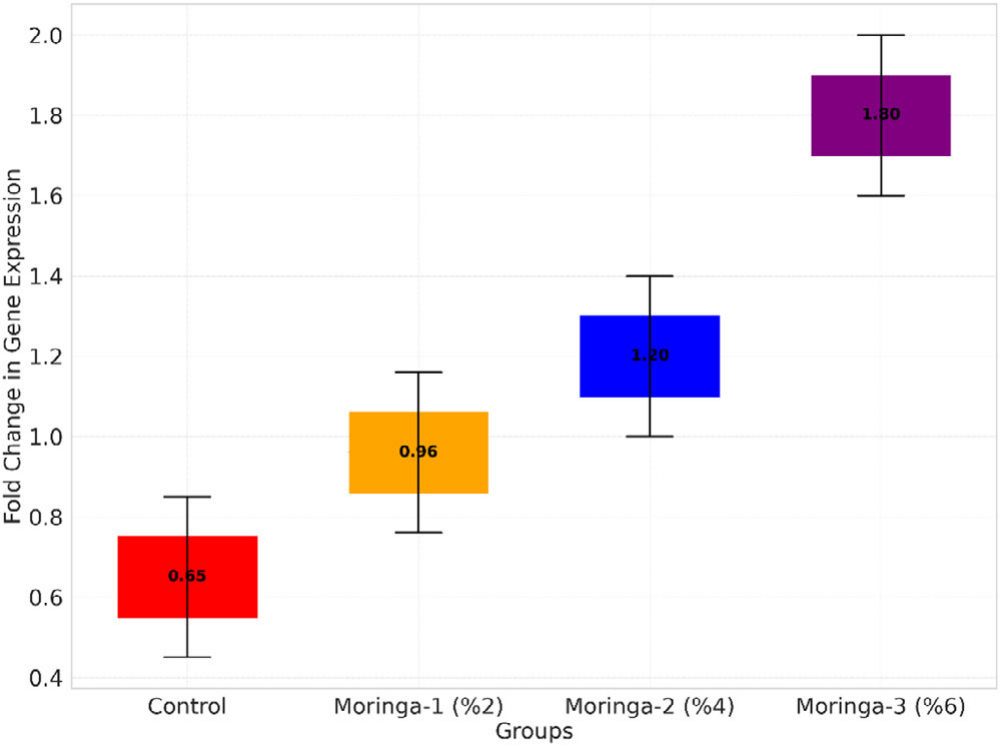
Expression levels of MYF5 gene.

**FIGURE 2:**
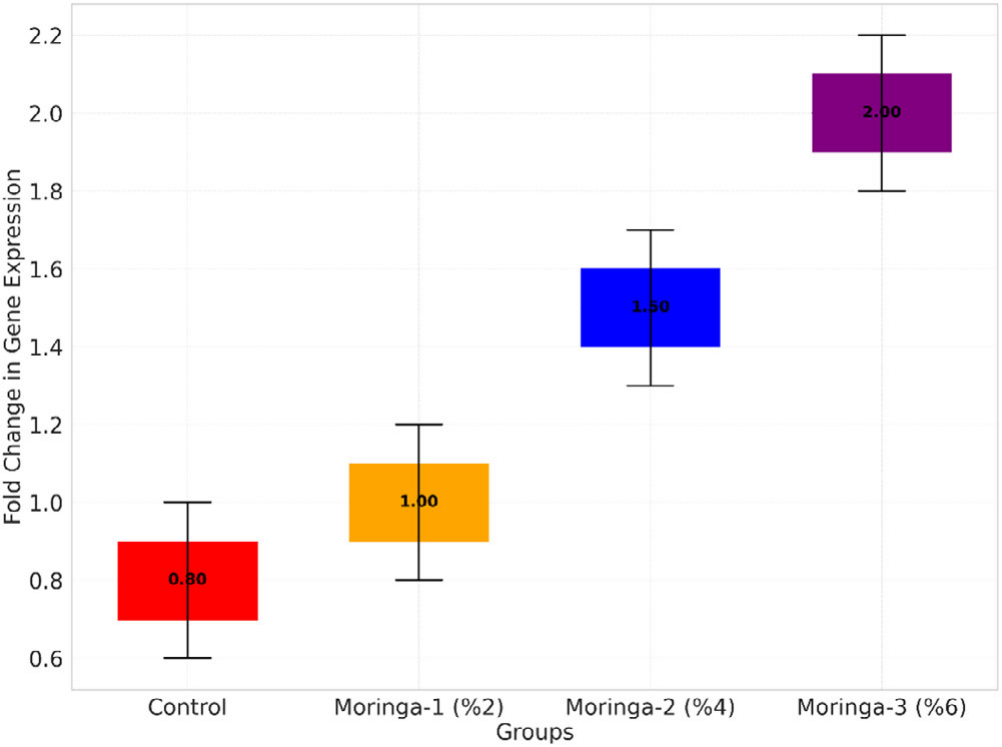
Expression levels of IGF‐1 gene.

The relative mRNA expression levels of MYF5 and IGF‐1 in breast muscle tissues are presented in Figure 1. The expression levels of MYF5 and IGF‐1 increased significantly (*p* < 0.05) with MOLP in the diet. The highest expression levels of both genes were observed in the Moringa‐3 group (6% MOL inclusion), with MYF5 and IGF‐1 expression levels reaching 1.80 and 2.0, respectively, compared to the Control group (0.65 and 0.80, respectively) (Figures 1, 2). The results indicate a dose‐dependent upregulation of MYF5 and IGF‐1, suggesting that the supplementation of MOLP positively influences muscle development through genetic regulation.

## 4 Discussion

The studies collectively demonstrate the potential of MOLP and its derivatives as feed additives to enhance the growth performance, health and productivity of poultry, particularly Japanese quail. Across these investigations, consistent themes emerge regarding the impact of MO on growth, carcass quality, blood biochemical profiles, antioxidant status, immune system, and egg production, albeit with some variations based on the form, dosage, and combination of MOL and other dietary supplements. Studies such as those by Elkloub et al. (2015), Mousa et al. (2016), and Minj et al. (2018) consistently show that incorporating MOL meal or germinated seed powder at moderate levels significantly improves body weight gain, feed conversion ratios (FCR), and production efficiency indices. For instance, Elkloub et al. (2015) highlighted that 0.2% MOL meal yielded the best FCR and European Production Efficiency Index (EPEI). Similarly, Mousa et al. (2016) found that 0.75% germinated MO seed powder provided the highest body weight gain and FCR. These findings suggest that MO supplementation optimises nutrient utilisation and metabolic efficiency, possibly due to its rich nutrient profile and bioactive compounds. Poultry feeds contain polyunsaturated fatty acids that can create reactive oxygen species (ROS) (Anonymous, 2025), and excessive production of ROS can cause poor nutrient absorption and digestion (Liu et al., 2014), leading to negative implications on performance traits in animals. MO contains antioxidant compounds that are known to neutralise reactive oxygen species (ROS) by giving up some of their own electrons, which acts as a natural ‘off’ switch for the free radicals. This helps break a chain reaction that can affect other molecules in the cell and other cells in the body, leading to the prevention of oxidative damage in poultry cells. In addition, Lü et al. (2010) also reported that antioxidants have the potential to decrease oxidative damages directly via reacting with free radicals or indirectly by inhibiting the activity or expression of free radical‐generating enzymes or enhancing the activity or expression of intracellular antioxidant enzymes. This reduction in the production of ROS inhibits the activation of the mechanism that is involved in the activation of oxidative stress in animals. However, as reported by Castillo et al. (2018) and Minj et al. (2018), excessive inclusion levels (>14% or 4.5%, respectively) can negatively affect weight gain and FCR, particularly during early growth phases.

The improvement in carcass yield and quality was a notable finding in several studies. Mousa et al. (2016) and Elkloub et al. (2015) reported significant reductions in abdominal fat and increased immune organ weights, indicating that MO reduces fat deposition while enhancing immune function. Similarly, Jalil and Yurtseven (2023) observed significant increases in carcass and liver weights with MO and canola seed supplementation. Maqsood et al. (2024) also reported improved carcass organoleptic properties and higher liver and heart weights with a mixed diet of MO and pomegranate peel powder, highlighting the synergistic potential of combining MO with other functional ingredients. Oxidative stress has been reported to decrease growth performance and nutrient digestibility (Yuan et al. 2007); however, MO is characterised by several bioactive compounds with antioxidant properties (Mahmoud et al. 2019; Castillo et al. 2018) capable of preventing oxidative stress and improving blood antioxidant enzyme levels. Improving the antioxidant status will in turn improve the immune system and the general welfare of animals with the subsequent improvement in feed intake, body growth and development. Additionally, MO is rich in fatty acids, and the addition of MO in diets could improve the fatty acid profile of meat as well as inhibit lipid oxidation and the development of oxidative rancidity in meat (Amaral et al. 2018). Mahmoud et al. (2019) observed significant upregulation of antioxidant genes (e.g., GSH‐Px, SOD and CAT) in broilers fed MO under heat stress, while Castillo et al. (2018) found reduced levels of ALT, AST and creatinine, especially at higher inclusion levels (21%).

Additionally, most studies, including those by Mousa et al. (2016) and Ashour et al. (2020), reported reduced cholesterol, triglycerides, and LDL levels and increased HDL levels. These effects underline MO's ability to enhance liver health and lipid metabolism, which may be attributed to its high polyphenol and flavonoid content. MO also positively impacts egg production and quality, as demonstrated by Abou‐Elkhair et al. (2020) and Ashour et al. (2020). Abou‐Elkhair et al. (2020) showed that 0.3% MO seed powder significantly increased egg production, egg yolk index, and reproductive gene expression (e.g., ESR2, FSHR and STAR). Ashour et al. (2020) corroborated these findings, reporting enhanced egg production, shell thickness, and hatchability with MO supplementation. These improvements are likely linked to MO's rich nutrient profile, which supports hormonal and reproductive functions. Several studies explored the combined effects of MO with other dietary components. Maqsood et al. (2024) found that combining MO with pomegranate peel powder enhanced carcass quality and ileum histology, while Jalil and Yurtseven (2023) reported synergistic benefits of MO and canola seed powder on growth performance, plasma proteins, and microbial balance. These results suggest that combining *Moringa oleifera* with other nutraceuticals can amplify its health‐promoting effects and provide additional benefits. MO's antimicrobial and immunomodulatory properties were highlighted in studies such as Castillo et al. (2018) and Jalil and Yurtseven (2023).

Castillo et al. (2018) demonstrated that *Moringa oleifera* inhibits gram‐positive and gram‐negative bacteria growth, while Jalil and Yurtseven reported a reduction in pathogenic bacteria in the digestive tract, improving digestive and immune health. These findings underscore MO's potential as a natural alternative to antibiotic growth promoters (AGPs). A recurring theme across studies is the importance of determining optimal inclusion levels of *Moringa oleifera*. While low to moderate levels (e.g., 0.2–0.75%) consistently yield positive results, excessive inclusion may lead to adverse effects, such as reduced weight gain or feed efficiency during certain growth stages, as noted by Castillo et al. (2018) and Minj et al. (2018). High levels of anti‐nutritional factors in the diet reduce digestibility by forming complexes with certain nutrients as well as damaging the digestive mucosa, leading to poor digestion and absorption of nutrients. Tannins, for instance, can injure the intestinal mucosa and interfere with the activity of digestive enzymes with the consequent decrease in digestibility and availability of amino acids and minerals. This emphasises the need for precise dietary formulations tailored to the specific requirements of poultry.

The collective findings highlight *Moringa oleifera*’s versatility as a natural, cost‐effective feed additive with multiple benefits for poultry production. Indeed, a higher selling price per carcass and higher gross margin in chickens supplemented with MO than those fed the control diet have been reported (Ayssiwede et al. 2011). Kairalla et al. (2023) also reported that the supplementation of 0.75% moringa powder in the broiler diet as a growth promoter reduces the cost of production.

Additionally, its ability to enhance growth, improve carcass quality, support reproductive performance, and mitigate heat stress and oxidative damage makes it a valuable tool in sustainable poultry nutrition. However, further research is needed to standardise optimal inclusion levels, explore long‐term effects, and evaluate its economic feasibility in commercial settings. Additionally, studies on its effects in combination with other functional ingredients, as demonstrated by Maqsood et al. (2024) and Jalil and Yurtseven (2023), offer promising avenues for maximising its potential in poultry diets. In conclusion, *Moringa oleifera* and its derivatives have shown consistent and significant benefits across various aspects of poultry production. They are a viable alternative to synthetic additives and a promising component of sustainable animal nutrition strategies.

## 5 Conclusions

This study demonstrates the significant potential of *Moringa oleifera* leaf as a natural feed additive to enhance growth performance and muscle development in Japanese quails. The inclusion of MOLP in the diet improved the body weight of quails, with optimal results observed at the inclusion levels of 4% and 6%. Additionally, MYF5 and IGF‐1 gene expression upregulation underscores *Moringa oleifera*’s role in modulating molecular pathways critical for muscle differentiation and growth. These findings align with and expand upon previous studies, further establishing *Moringa oleifera* as a versatile and effective dietary supplement. The results highlight its potential to optimise nutrient utilisation, improve metabolic efficiency, and support sustainable poultry production. However, anti‐nutritional factors in *Moringa oleifera* necessitate carefully considering inclusion levels and appropriate processing methods to maximise its benefits while minimising adverse effects. Future research should focus on long‐term studies to evaluate the economic feasibility and commercial scalability of *Moringa oleifera* supplementation. Investigations into its synergistic effects with other functional feed ingredients and its impact on carcass quality, reproductive performance, and overall poultry health are also warranted. By integrating genetic and nutritional approaches, *Moringa oleifera* can be crucial in advancing sustainable and efficient poultry nutrition strategies.

## Author Contributions

**Kadriye Kurşun**: conceptualisation, methodology, writing – review and editing, visualization, writing – original draft, formal analysis, data curation, resources, project administration, validation, investigation, software, supervision and funding acquisition.

## Ethics Statement

This study was conducted under the guidelines for animal experiments of the Ministry of Food, Agriculture and Livestock, Türkiye. Approval was granted by the local ethics committee of Cukurova University **(Approval number: No 1/31.01.2025)**.

## Conflicts of Interest

The author declares no conflicts of interest.

### Peer Review

The peer review history for this article is available at https://www.webofscience.com/api/gateway/wos/peer‐review/10.1002/vms3.70400.

## Data Availability

The data for this study is available with the corresponding author and will be shared upon request.
